# Current Status and Future Trends in *Campylobacter* Detection and Quantification Across the Food Production Continuum

**DOI:** 10.3390/pathogens15101024

**Published:** 2026-09-30

**Authors:** Varsha Bommineni, Lekshmi K. Edison, Subhashinie Kariyawasam

**Affiliations:** Department of Comparative, Diagnostics, and Population Medicine, University of Florida, Gainesville, FL 32610, USA; vbommineni@ufl.edu (V.B.); edison.le@ufl.edu (L.K.E.)

**Keywords:** *Campylobacter jejuni*, *Campylobacter coli*, food safety, detection, quantification, qPCR, digital PCR, viable-but-non-culturable, whole-genome sequencing, One Health surveillance

## Abstract

*Campylobacter* spp., particularly *Campylobacter jejuni* and *Campylobacter coli*, remain the leading bacterial causes of foodborne gastroenteritis worldwide, with poultry serving as the dominant reservoir and additional transmission routes involving livestock, raw milk, water, fresh produce, and food-processing environments. Accurate detection and quantification are essential for risk assessment, process hygiene monitoring, outbreak investigation, and One Health surveillance; however, reliable measurement is complicated by the organism’s fastidious microaerophilic growth requirements, stress sensitivity, low or uneven distribution in complex matrices, and ability to enter viable but non-culturable (VBNC) states. This review examines current and emerging approaches for *Campylobacter* detection and quantification across the food production continuum, including pre-harvest, harvest and processing, post-harvest, and retail, as well as consumer-level settings. Conventional culture, colony-count enumeration, most probable number (MPN) methods, and immunological assays are compared with molecular approaches, including polymerase chain reaction (PCR), qPCR, digital PCR, viability PCR, whole-genome sequencing, metagenomics, biosensors, microfluidics, and artificial intelligence-assisted surveillance. Regulatory and standardization frameworks, including the International Organization of Standards (ISO), Food and Drug Administration (FDA), United States Department of Agriculture-Food Safety and Inspection Service (USDA-FSIS), and European Union (EU) process hygiene criteria are discussed, together with One Health surveillance systems. Overall, future *Campylobacter* monitoring will require integrated, matrix-specific strategies that combine standardized culture-based enumeration, viability-informed molecular quantification, genomic source attribution, and harmonized metadata to support more rapid, accurate, and risk-based food-safety decision-making.

## 1. Introduction

Unsafe food is now recognized not as an episodic problem but as a persistent global public health and food system challenge. Recent global estimates indicate that unsafe food causes approximately 866 million cases of illnesse and 1.5 million deaths annually, with young children carrying a disproportionate share of the burden [1]. Within this broader context, *Campylobacter* represents a major bacterial foodborne pathogen worldwide. *Campylobacter* is recognized as one of the four major global causes of diarrheal disease and is considered the most common bacterial cause of human gastroenteritis, with the thermotolerant species *C. jejuni* and *C. coli* most frequently associated with human disease [2]. The epidemiology of campylobacteriosis further illustrates why detection and quantification are critical across the food production continuum. In the United States, infection is most commonly associated with the consumption of raw or undercooked poultry, although contaminated foods, untreated water, and contact with animals may also contribute to transmission [3,4]. In Europe, campylobacteriosis remains the most frequently reported zoonotic disease in the European Union (EU), highlighting its sustained public health importance [5]. Collectively, these data emphasize the need for sensitive, standardized, and quantitative detection strategies to monitor *Campylobacter* across farm, processing, retail, and consumer-level stages of the food chain.

The genus *Campylobacter* comprises spiral, curved, or S-shaped Gram-negative, microaerophilic bacteria that are widely distributed among warm-blooded animals, particularly poultry, cattle, pigs, sheep, and companion animals [2]. Among clinically important species, *C. jejuni* is the predominant cause of human disease; the Centers for Disease Control and Prevention (CDC) reports that nearly 90% of human *Campylobacter* infections is caused by *C. jejuni*, while *C. coli* and other species account for a smaller proportion of cases [3]. This predominance is highly relevant to food-chain surveillance because both species are well adapted to animal reservoirs, especially poultry, where intestinal colonization is often asymptomatic but can lead to substantial contamination of carcasses or products during slaughter, processing, and food preparation [2,4]. Antimicrobial resistance (AMR) has also become an increasingly important component of *Campylobacter* surveillance because resistance patterns vary by species, lineage, host reservoir, and geography. Surveillance systems such as the National Antimicrobial Resistance Monitoring System (NARMS) track resistance in *Campylobacter* from humans, retail meats, and food animals, supporting a One Health approach to understanding of resistant foodborne infections [6].

Transmission of *Campylobacter* occurs primarily through foodborne routes, although non-foodborne pathways also contribute to human exposure. The World Health Organization (WHO) identifies undercooked meat and meat products, raw or contaminated milk, contaminated water or ice, recreational water exposure, and contact with animals or animal products as importantroutes of transmission [2]. Poultry remains the dominant reservoir in many settings. The EU regulatory framework cites evidence that handling, preparation, and consumption of broiler meat may directly account for approximately 20–30% of human campylobacteriosis cases, while 50–80% of cases may be attributable to the chicken reservoir more broadly; this evidence underpins the EU process hygiene criterion for *Campylobacter* on broiler carcasses [7]. Sequence-based source-attribution studies have consistently supported the central role of poultry, particularly chicken, as a major reservoir for human campylobacteriosis. In a population-genetic analysis of 1,231 human *C. jejuni* cases from Lancashire, England, most sporadic infections were attributed to livestock and poultry reservoirs, with chicken identified as the leading source, followed by cattle [8]. Subsequent genome-based analyses further reinforced the contribution of chicken to human infection, while also highlighting important limitations in source attribution. In particular, host switching, strain sharing among animal reservoirs, and incomplete sampling of potential transmission pathways can weaken host-specific genomic signals and complicate precise attribution of human cases [9]. Therefore, although poultry should be regarded as a primary reservoir and priority target for intervention, surveillance frameworks should also consider additional animal, environmental, and food-chain pathways that may contribute to human exposure.

Dairy and waterborne transmission require separate emphasis because their epidemiological patterns differ from poultry-associated sporadic disease. Raw or unpasteurized milk is a well-recognized vehicle for *Campylobacter* outbreaks, and the CDC warns that raw milk can expose consumers to several pathogens, including *Campylobacter*, even when farms follow good hygiene practices [10]. Waterborne transmission is more difficult to quantify as a stable fraction of overall disease burden, but it remains epidemiologically important, particularly in localized outbreaks involving contaminated drinking-water systems. Large waterborne outbreaks, including those reported in Norway and New Zealand, illustrate how contamination of drinking-water sources can generate substantial community-level exposure [11,12]. Collectively, the evidence supports a hierarchical rather than monocausal model of transmission: poultry is a principal reservoir for human campylobacteriosis, but dairy, water, environmental exposure, and direct animal contact remain important outbreak-associated and geographically variable pathways that must be considered in food-chain detection and quantitative surveillance.

*Campylobacter* detection based solely on presence/absence testing is insufficient, as public health risk depends strongly on the number of viable organisms reaching consumers. Dose–response models show that probability of infection is influenced by ingested dose, exposure route, food matrix, and host susceptibility; therefore, two positive samples may pose very different risks depending on their bacterial loads [4,13]. Quantification is therefore a core element of exposure assessment, risk characterization, and evidence-based food-safety decision-making. This principle is reflected in EU regulation, which established a process hygiene criterion for *Campylobacter* on broiler carcasses after chilling, based on enumeration according to EN ISO 10272-2 [14] and a limit of 1000 CFU/g. Compliance with this limit was expected to substantially reduce the public health risk associated with broiler meat consumption by more than 50% [7,15]. Unlike prevalence data alone, quantitative enumeration captures the distribution of contamination levels across batches, carcasses, and servings, allowing thresholds to be more directly linked with predicted consumer risk [16].

Together, these considerations show that *Campylobacter* detection across the food production continuum must move beyond simple presence/absence testing toward approaches that also quantify viable bacterial burden and support source tracking. Culture-based enumeration remains essential for regulatory monitoring and international standardization; however, it may underestimate contamination when *Campylobacter* cells are stressed, sublethally injured, or enter viable but non-culturable (VBNC) states. Viability-informed molecular approaches, including propidium monoazide quantitative polymerase chain reaction (PMA-qPCR) and propidium monoazide combined with droplet digital PCR (PMA-ddPCR), provide faster and more viability-aware quantification, while digital PCR enables absolute quantification without standard curves [15]. Despite these advances, an important research gap remains: current detection systems are not yet fully harmonized for routine, stage-specific, viability-aware, and quantitative monitoring across diverse food matrices and production environments. In particular, limited standardization between culture-based and molecular methods, variability in sample preparation, and incomplete integration of quantitative data with genomic source-tracking continue to limit risk-based surveillance. Therefore, future monitoring will likely depend on integrated strategies that combine standardized culture-based enumeration, molecular quantification, and genomic typing. In this context, the present review examines current and emerging methods for *Campylobacter* detection and quantification across the farm-to-fork continuum, highlighting their applications, limitations, research gaps, and future role in food-safety monitoring and public-health decision-making. Previous reviews [17,18,19,20,21,22,23,24] have addressed specific aspects of *Campylobacter* detection, including culture-based methods, molecular approaches, biosensors, and surveillance; a summary of their scope and key areas of focus is provided in Supplementary File S1.

## 2. Biology and Ecology of *Campylobacter* Relevant to Detection

The biological characteristics of *Campylobacter* are central to understanding why its detection and quantification remain challenging across the food production continuum. Members of the genus are small, curved, spiral, or S-shaped Gram-negative bacteria, commonly described as slender rods with characteristic corkscrew motility. The most clinically important species, particularly *C. jejuni* and *C. coli*, are widely distributed in warm-blooded animals and are especially associated with poultry and other livestock reservoirs [2,25]. Their non-fermentative metabolism, reliance on amino acids and tricarboxylic acid cycle intermediates, and limited carbohydrate utilization further distinguish them from many other enteric foodborne pathogens and contribute to their fastidious growth requirements [25,26,27]. These traits are important for detection because the organism is often present in complex matrices such as feces, carcass rinses, poultry skin, water, milk, and environmental samples, where competing microbiota and matrix inhibitors can reduce analytical sensitivity.

Thermotolerant *Campylobacter* species are fastidious and microaerophilic, requiring reduced oxygen tension for optimal growth. *C. jejuni* grows best at approximately 37–42 °C, which is consistent with its adaptation to avian hosts, while growth below 30 °C is generally poor or absent [3,25]. Standard culture methods therefore require selective enrichment, microaerobic incubation, and selective media that suppress competing organisms while supporting recovery of *Campylobacter*. However, these same selective conditions may inhibit injured or physiologically stressed cells, increasing the risk of underestimation or false-negative results in samples exposed to refrigeration, oxygen, drying, freezing, low pH, prolonged storage, or other food-processing stresses [2,25,28].

A major detection challenge is *Campylobacter*’s ability to enter a VBNC state under unfavorable environmental conditions. In this state, cells may remain metabolically active or potentially recoverable but fail to form colonies on routine culture media. Stressors such as cold exposure, oxidative stress, nutrient limitation, and other processing-related conditions can reduce culturability without necessarily eliminating viable cells [15,25]. This has important implications for food-safety surveillance because culture-based enumeration may underestimate the viable or potentially recoverable populations, especially in chilled poultry, carcass rinses, and other matrices where cells may be injured or stressed. Molecular methods such as PMA-qPCR and PMA-ddPCR are therefore increasingly investigated as viability-informed tools to detect intact cells that may be overlooked in conventional culture, while reducing signals from dead cells [15]. Experimental in vivo studies indicate that at least some non-culturable *C. jejuni* populations can regain culturability following passage through biological hosts. For example, Cappelier et al. [29] recovered VBNC *C. jejuni* after inoculation into embryonated eggs, with the recovered cells retaining the ability to adhere to HeLa cells. Similarly, Baffone et al. [30] reported restoration of culturability following passage of VBNC *C. jejuni* through the murine intestine. In contrast, another study [31] was unable to recover non-culturable *Campylobacter* from experimentally challenged chicks or mice. Thus, evidence for in vivo resuscitation remains inconsistent. In addition, the direct infectivity of VBNC *Campylobacter* in humans has not been established.

Although *Campylobacter* is often described as fragile outside the host, it can persist in the food chain and environmental settings through stress adaptation, surface association, and survival within mixed microbial communities. The CDC noted that *Campylobacter* is fragile outside the host and sensitive to drying, while freezing reduces but does not necessarily eliminate contamination on raw meat. Experimental and review evidence further indicates that survival is influenced by temperature, water activity, pH, oxygen exposure, humidity, and the composition of the surrounding matrix. For example, *Campylobacter* grows optimally near neutral pH; is inhibited at more acidic or alkaline pH values; and is sensitive to heat, desiccation, and oxidative stress, although survival can be prolonged under refrigerated or protective matrix conditions [3,25,28,32]. These features mean that negative culture results may reflect poor recovery rather than true absence, particularly when samples subjected to refrigeration, freezing, drying, or disinfectant exposure.

Biofilm formation and surface persistence further complicate detection. *Campylobacter* can attach to food-contact and environmental surfaces, and its survival may be enhanced when cells are embedded in biofilms or associated with other microorganisms. Biofilm-associated cells can be unevenly distributed, more resistant to environmental stress, and harder to detach during sampling, which may reduce detection sensitivity and contribute to inconsistent recovery from processing equipment, water systems, and poultry-processing environments [33,34,35]. Therefore, sampling strategy, surface swabbing efficiency, enrichment conditions, and the choice of culture or molecular method are all critical determinants of detection performance.

Overall, the ecology of *Campylobacter* creates a paradox for food-safety monitoring: the organism is fastidious and environmentally sensitive, yet it remains highly successful in transmission through poultry, animal reservoirs, water, milk, and food-processing environments. Its microaerophilic growth requirements, stress sensitivity, potential VBNC transition, surface association, and matrix-dependent survival can all reduce culture recovery and contribute false-negative or underestimated results. Consequently, reliable detection requires methods that account not only for presence or absence but also for viability, physiological state, bacterial load, sample matrix, and stage of the food production continuum [14,28].

## 3. Sources and Transmission Across the Food Production Continuum

**Figure 1:**
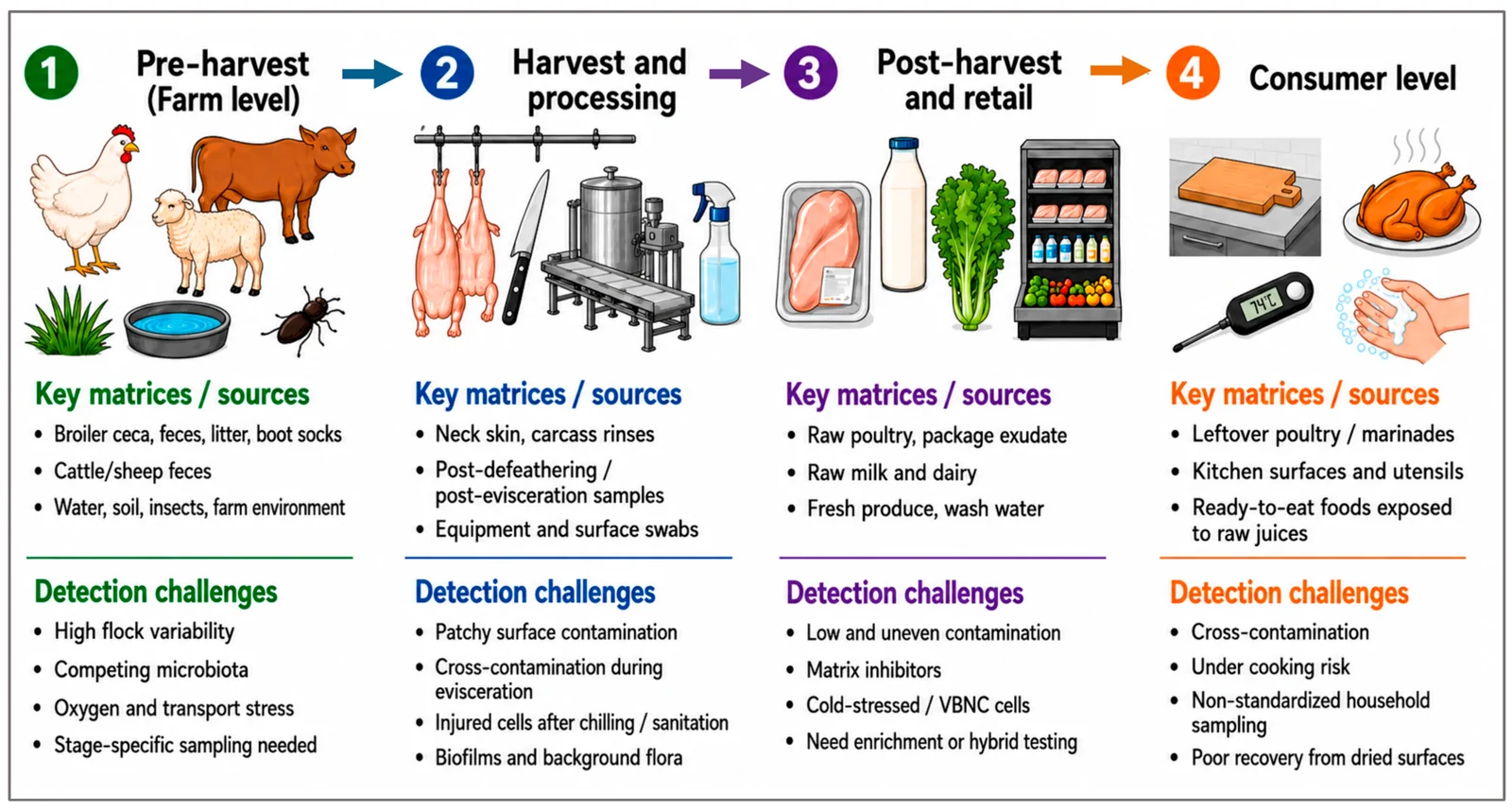
Farm-to-fork transmission and detection framework for *Campylobacter*. The schematic summarizes major *Campylobacter* sources, sample matrices, and detection challenges across four stages of the food production continuum: pre-harvest, harvest and processing, post-harvest and retail, and consumer-level stages. It highlights how bacterial load, matrix complexity, background microbiota, cell injury, VBNC transition, cross-contamination, and sampling strategy influence detection across the food production continuum (Created in BioRender. Bommineni, V. (2026) https://BioRender.com/uk9iea8).

*Campylobacter* detection is difficult because the organism is fastidious and oxygen-sensitive, and may be present at low levels, unevenly distributed, or physiologically stressed in food-chain samples. Therefore, detection and quantification strategies must be tailored to each stage of the food production continuum—the pre-harvest (farm level), harvest and processing, post-harvest and retail, and consumer-level stages—while accounting for differences in sample type, bacterial load, cell viability, and background microbiota. The farm-to-fork transmission routes, key sample matrices, and stage-specific detection challenges associated with *Campylobacter* are summarized in Figure 1.

### 3.1. Pre-Harvest (Farm Level)

At the pre-harvest stage, poultry, especially broiler chickens, represents the major reservoir of foodborne *Campylobacter* transmission. Thermotolerant species such as *C. jejuni* and *C. coli* colonize the avian intestinal tract, particularly the ceca, often at high loads without causing obvious disease in birds. This makes intestinal carriage a key upstream source of carcass contamination during slaughter. Other livestock, including cattle, sheep, and pigs, also serve as reservoirs, while farm-associated sources such as water, litter, dust, insects, rodents, personnel, equipment, and nearby wildlife may contribute to flock colonization and environmental spread [2,3,36,37]. Detection at the farm level is complicated by differences among sample matrices . Cecal and fresh fecal samples may contain high *Campylobacter* loads but also large amount of organic material and competing microbiota. In contrast, environmental samples such as water, soil, dust, litter, insects, and farm swabs often contain lower, intermittent, or stressed populations exposed to oxygen, drying, temperature changes, and nutrient limitation. These conditions can reduce culturability or promote VBNC states, causing culture-based surveillance to underestimate true prevalence [28,38].

Therefore, pre-harvest surveillance should use layered sampling rather than a single matrix. Cecal contents, cloacal or fecal samples, boot socks, and drag swabs are useful for flock-status assessment, while litter, dust, drinker-line water, house swabs, insect traps, and nearby livestock or environmental samples can help identify potential routes of introduction and dissemination. This approach is consistent with ISO 10272-2, which includes primary-production samples such as feces, dust, and swabs within its analytical scope [14]. Method selection should reflect expected bacterial load: direct culture and enumeration may be suitable for high-load cecal or fecal samples, whereas low-load or stressed environmental matrices usually require selective enrichment and, where appropriate, molecular or viability-informed assays. Pre-harvest control should therefore focus on multihurdle biosecurity, including pest control, water protection, litter and manure management, hygiene barriers, and reduced contact with adjacent livestock or contaminated runoff [39,40].

Seasonal and climatic associations with *Campylobacter* occurrence have been reported across farm and broiler production studies, although the patterns of these associations vary by geographical region, production setting, and sample type. Farm-level studies have reported higher *Campylobacter* prevalence in chicken and cattle fecal samples during rainy and cold periods, although the same seasonal pattern was not observed in drinking water [41]. In longitudinally monitored broiler farms, *Campylobacter* colonization was more likely under higher minimum indoor temperatures and higher minimum outdoor relative humidity, despite the absence of a clear overall seasonal pattern [42]. Another broiler study found that colonization risk increased with increasing cumulative temperature exposure during the weeks preceding slaughter [43]. Seasonal variation has also been reported in Great Britain, where *Campylobacter*-positive broiler flocks were more common from June to November, and flock prevalence was associated with temperature, rainfall, and sunshine duration [44]. Collectively, these findings suggest that climatic effects on *Campylobacter* occurrence are context-dependent and may differ substantially across locations, production environments, and sample types.

### 3.2. Harvest and Processing

The harvest and processing stage represents a major transition point between intestinal carriage in live birds and consumer exposure through contaminated meat. During slaughter, *Campylobacter* contamination occurs primarily through fecal transfer, with defeathering and evisceration recoganized as critical steps because externally contaminated birds and intestinal contents can spread organisms to carcass skin, equipment, and processing surfaces [2,45]. Stainless-steel contact surfaces, conveyors, knives, and cutting-stage equipment may serve as recurrent contamination nodes, allowing organisms from highly colonized flocks to be redistributed among carcasses and the processing environment. From a detection perspective, this stage is challenging because processing both redistributes and physiologically modifies *Campylobacter* cells. Scalding, rinsing, chilling, antimicrobial treatments, drying, and sanitation can reduce bacterial loads, but they may also injure surviving cells and suppress culturability. As a result, contamination patterns are often heterogenous and stage-dependent rather than uniform. Recent abattoir studies show that *Campylobacter* loads may decline overall during processing, yet post-defeathering, post-evisceration, and post-chill samples represent different contamination and recovery states [46].

Sampling design is therefore critical. Neck-skin excision is highly informative and is widely used for process hygiene monitoring, whereas whole-carcass rinses provide a broader estimate of carcass surface contamination. However, these approaches are not interchangeable, as neck-skin samples may yield higher mean *Campylobacter* counts than whole-carcass rinses [47]. For process mapping, sampling should include key points such as post-defeathering, post-evisceration, post-wash/decontamination, and post-chill, along with environmental swabs from plucking fingers, shackles, conveyors, knives, tables, drains, and other food-contact surfaces. A hybrid analytical strategy is most appropriate at this stage. Culture-based enumeration remains essential for regulatory monitoring and quantitative trend analysis, with ISO 10272-2 providing the reference colony-count framework and the Food and Drug Administration Bacteriological Analytical Manual (FDA BAM) offering practical guidance for enrichment and isolation [14,28]. Presumptive colonies should be confirmed by species-specific PCR, matrix-assisted laser desorption/ionization time-of-flight mass spectrometry (MALDI-TOF MS), or equivalent methods. When stressed or injured cells are expected, particularly after chilling or decontamination, viability PCR methods such as PMA-qPCR or PMA-ddPCR can complement culture by detecting viable cells while reducing dead-cell DNA signals [15]. Rapid enrichment-PCR workflows may also shorten turnaround time and improve process-control decisions [48].

Biofilm formation and surface persistence further complicate detection and control. Slaughterhouse-associated *Campylobacter* isolates can adhere to stainless steel and survive better in the presence of chicken exudate, while biofilm-associated cells may be harder to detach during swabbing and more tolerant of sanitation procedures [49,50]. Therefore, environmental monitoring should include difficult-to-clean niches and should be interpreted as part of a longitudinal process trending rather than as isolated one-time results. Overall, control at the harvest and processing stage depends on minimizing gut rupture and fecal leakage, optimizing defeathering and evisceration conditions, maintaining equipment hygiene, validating carcass interventions, and tracking quantitative results over time. Even effective processing interventions may fail to meet process-hygiene targets when incoming flock burden is high or when persistent environmental contamination is not controlled.

### 3.3. Post-Harvest and Retail

At the post-harvest and retail stages, raw poultry meat remains the dominant vehicle for *Campylobacter* exposure, while raw milk and fresh produce represent lower-frequency but important transmission routes. The WHO and CDC identify raw or undercooked poultry, raw or contaminated milk, untreated water, and contaminated produce as relevant sources of human exposure [2,4,10]. Detection challenges vary considerably by matrix. In chilled poultry meat, refrigeration, oxygen exposure, packaging conditions, and storage may injure cells or reduce culturability. The FDA BAM notes that samples should be analyzed as soon as a package is opened because fresh oxygen can further stress weakened *Campylobacter* cells [28]. In raw milk, contamination may be low and intermittent, requiring enrichment and careful handling before culture [51,52]. In fresh produce, *Campylobacter* contamination is usually less frequent than in poultry, but detection can be difficult because cells may be unevenly distributed and masked by abundant background flora on the plant surface or in the wash environment [53,54]. Sampling and testing should therefore be adapted to the product type. For retail poultry, useful matrices include skin-on parts, neck skin, package exudate, carcass rinses, and composite samples across lots or product forms [55,56]. For raw milk, testing may need to include not only milk but also teat swabs, milk filters, milking equipment, and fecal or environmental sources when contamination routes are being investigated [57,58]. For fresh produce, edible portions, rinsates, wash water, irrigation water, and processing-environment samples may be required, especially when source attribution is the goal [54].

A hybrid laboratory approach is most appropriate for post-harvest and retail surveillance. Culture-based methods remain essential for obtaining viable isolates for confirmation, antimicrobial susceptibility (AST) testing, or genomic source attribution; however, enrichment is often necessary for low-level or stressed samples. For chilled poultry and other matrices where injured or VBNC cells may be present, viability PCR methods such as PMA-qPCR or PMA-ddPCR can complement culture by improving detection of viable cells while reducing dead-cell DNA signals [14,15]. Control priorities at this stage are product-specific. For poultry meat, maintaining the cold chain, preventing package leakage, and reducing cross-contamination during storage and handling are critical. For dairy, pasteurization remains the most effective control measure, as hygienic milking alone cannot fully eliminate the risk of intermittent contamination. For fresh produce, prevention depends on safe agricultural water, proper manure management, hygienic control of wash water, and separation from raw animal products during retail handling and storage [59,60].

### 3.4. Consumer Level

At the consumer level, *Campylobacter* transmission occurs mainly through undercooked poultry and cross-contamination in the kitchen. The CDC notes that raw or undercooked poultry is a major source of infection and that even a small amount of raw chicken juice can contain enough *Campylobacter* to contaminate ready-to-eat foods, cutting boards, knives, and other kitchen surfaces [4]. Therefore, poultry should be cooked to an internal temperature of 165 °F (74 °C); raw meat should be kept separate from ready-to-eat foods; and cutting boards, utensils, countertops, and hands should be cleaned thoroughly after handling raw poultry. Washing raw poultry should be avoided because it can spread bacteria through splashing and increase kitchen cross-contamination [61,62].

Detection at the consumer stage is less standardized than surveillance at the farm, processing, or retail stages. Household investigations usually rely on outbreak- or research-specific sampling of leftover poultry; marinades; drip-contaminated foods; and swabs from sinks, taps, cutting boards, utensils, cloths, and handles. However, recovery may be inconsistent because household surfaces expose cells to drying, oxygen, detergents, and time delays before sampling. Culture remains important when viable isolates are required, but molecular methods, including conventional PCR or viabilty PCR may help when stressed cells are suspected and culture results are negative [14,15,28]. Thus, consumer-level prevention depends on simple but critical behaviors: washing of raw poultry, separating raw and ready-to-eat foods, washing hands and utensils after raw-meat handling, refrigerating foods promptly, and using a thermometer rather than visual appearance to confirm cooking. These practices represent the final barrier to exposure, but they cannot replace upstream control because the contamination entering the kitchen are influenced by pre-harvest colonization, slaughter hygiene, processing, and retail handling.

## 4. Conventional Detection and Quantification Methods for *Campylobacter*

Detection and quantification of *Campylobacter* remain challenging because the organism is fastidious, microaerophilic, and susceptible to physiological injury outside the host. Conventional culture, therefore, remains the regulatory reference for food-chain testing, with ISO 10272-1:2017 [63] guiding detection, ISO 10272-2:2017 guiding colony-count enumeration, and FDA BAM supporting enrichment, isolation, and confirmation [14,28]. Although culture confirms the viability of culturable organisms and provides isolates for further characterization, it is time-consuming and may underestimate the number of stressed or VBNC cells. MPN methods support viable estimation in low-level or complex matrices but are labor-intensive, whereas enzyme-linked immunosorbent assay (ELISA) and lateral-flow assays provide rapid screening but remain adjunctive due to matrix-dependent sensitivity, potential cross-reactivity, and the need for culture confirmation.

### 4.1. Culture-Based Methods

**Figure 2:**
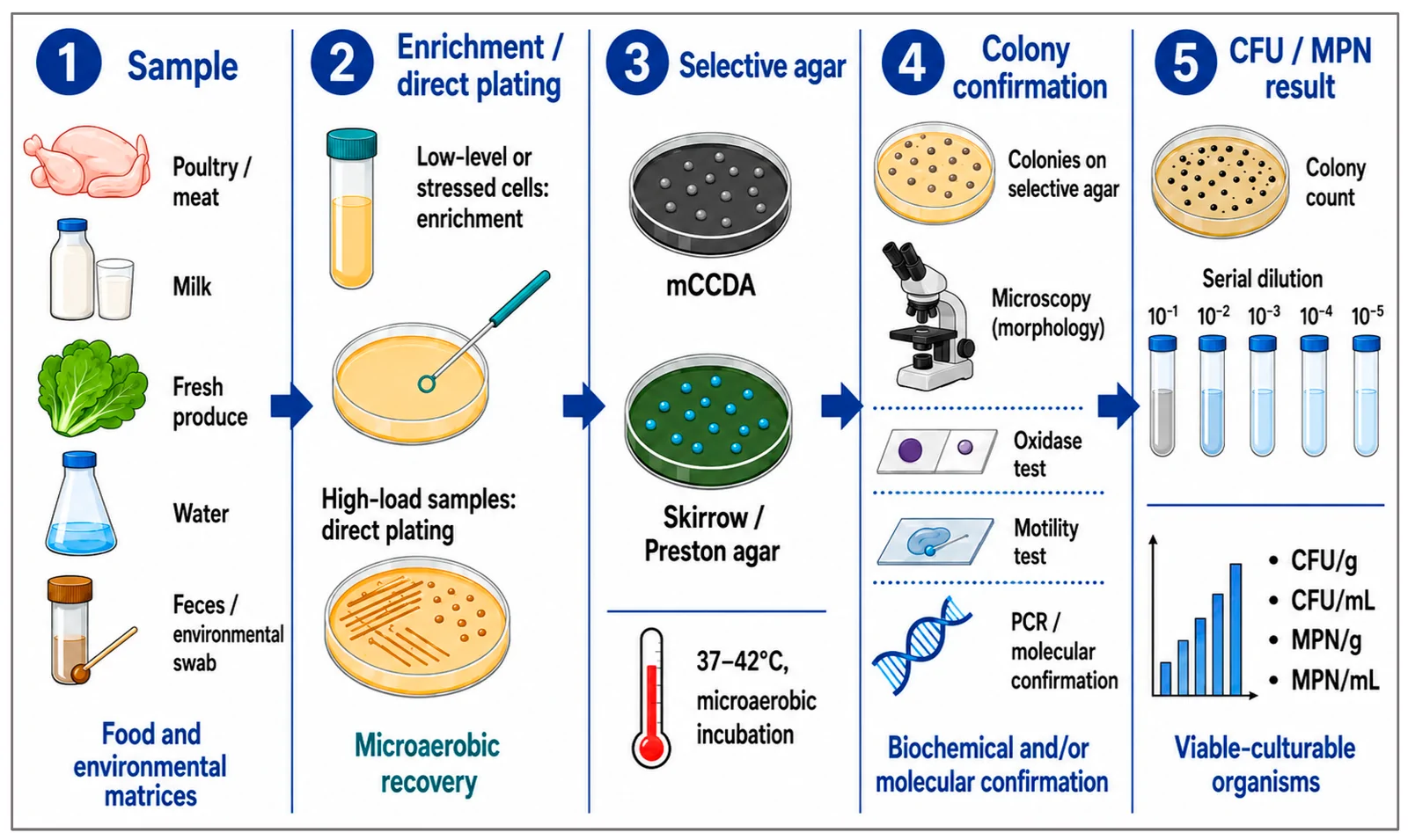
Conventional culture-based workflow for *Campylobacter* detection and quantification. Samples from food or environmental matrices are processed by selective enrichment or direct plating, followed by inoculation on selective agar (mCCDA: modified charcoal cefoperazone deoxycholate agar), colony confirmation, and interpretation of results as colony-forming units (CFU) or most probable number (MPN). The workflow highlights the major strengths of culture-based methods, including the detection of viable organisms and the recovery of isolates for regulatory and confirmatory testing, as well as important limitations, such as slow turnaround time, reduced sensitivity in complex matrices, and failure to detect viable-but-non-culturable (VBNC) cells (Created in BioRender. Bommineni, V. (2026) https://BioRender.com/jcp4ljf).

Culture-based detection remains the reference approach for *Campylobacter* because it links a positive result to recovery of viable, culturable organisms and provides isolates for confirmation, AST testing, and molecular characterization. In food-chain testing, the workflow generally follows either direct plating for high-load matrices or selective enrichment followed by plating when contamination is low or unevenly distributed, or cells are likely to be injured. ISO 10272-1:2017 provides the horizontal detection framework by enrichment or direct plating, whereas ISO 10272-2:2017 formalizes colony-count enumeration; FDA BAM Chapter 7 provides practical guidance for sample preparation, enrichment, isolation, incubation under microaerobic conditions, and confirmation [14,28]. The conventional culture-based workflow used for *Campylobacter* detection and quantification is summarized in Figure 2.

Selective recovery is central to culture-based testing because *Campylobacter* grows slowly and is easily overgrown by background microbiota. Standard workflows typically use microaerobic atmospheres, commonly 5% O_2_, 10% CO_2_, and 85% N_2_, with incubation at 37–42 °C, depending on the recovery step and target species [22,28]. For stressed cells, pre-enrichment or step-wise recovery procedures can improve culturability before exposure to stronger selective conditions. This is especially important for chilled poultry, carcass rinses, raw milk, produce, and environmental samples, where cells may be sublethally injured by oxygen, refrigeration, drying, sanitation, or other matrix-associated stress.

The development of selective media has shaped modern *Campylobacter* culture. Skirrow’s medium first enabled the practical isolation of thermotolerant *Campylobacter* from feces using antibiotic-based suppression of competing microbiota [64]. Preston medium later improved selectivity for animal and environmental specimens, and Preston enrichment further enhanced recovery compared with direct plating in some matrices [65,66]. Blood-free charcoal-based media were then developed to reduce oxidative stress and support recovery without blood supplementation, forming the basis for modified charcoal cefoperazone deoxycholate agar (mCCDA), which remains widely used in ISO-aligned food microbiology [14,66,67]. Despite its importance, selective culture is not analytically neutral. Recovery depends strongly on the enrichment broth, selective agar, antimicrobial supplement, competing flora, and physiological condition of the target cells. For example, modification of mCCDA with higher polymyxin B improved selectivity for chicken carcass rinses, while alternative enrichment–plating combinations have improved detection from chicken meat in some studies [68,69]. Background organisms, particularly ESBL-producing *E. coli*, can compromise selectivity by growing on cefoperazone-based media, prompting evaluation of broth or agar modifications with β-lactamase inhibitors or other supplements [69,70,71].

For quantification, CFU-based colony counting remains most useful in high-load matrices such as broiler ceca, feces, neck skin, and some carcass rinses. Its main advantage is interpretability: confirmed colonies are directly converted to CFU/g, CFU/cm^2^, or CFU per sample, making the method suitable for process hygiene monitoring and regulatory comparisons [14,28]. Culture also provides isolates for biochemical confirmation, species-specific PCR, MALDI-TOF MS identification, AST testing, and genomic source attribution. However, culture-based methods have important limitations. They are time-consuming, often requiring 48–72 h or longer when enrichment, subculture, and confirmation are included. More importantly, they detect only culturable cells. *C. jejuni* can enter non-culturable coccoid or VBNC states under stress, meaning that culture may underestimate viable populations in chilled, oxygen-exposed, disinfected, or nutritionally stressed samples [72,73]. Therefore, culture remains indispensable for regulatory confirmation and isolate recovery, but it should be interpreted as a measure of viable and culturable *Campylobacter* rather than total biological presence.

### 4.2. Most Probable Number (MPN)

The most probable number (MPN) method is a viable cell enumeration approach used when direct plating is insufficiently sensitive or unsuitable for a given matrix. Instead of counting colonies directly, serial dilutions of a sample are inoculated into replicate broth tubes or wells, incubated, and scored as positive or negative. The bacterial concentration is then estimated statistically based on the pattern of positive reactions across dilutions. FDA BAM Appendix 2 describes MPN as particularly useful for low organism concentrations, especially below approximately 100 organisms/g, and for matrices such as milk, water, and particulate foods that may interfere with accurate colony counting [74]. For *Campylobacter*, MPN is most useful when viable organisms are expected at low levels or when direct plating is affected by matrix interference. Relevant applications include retail poultry rinses with low counts, raw milk, environmental water, and selected farm or slaughterhouse surveillance samples. Miniaturized *Campylobacter*-specific MPN formats have also been developed to reduce media use and improve practicality. For example, Chenu et al. developed a miniaturized MPN method for thermophilic *Campylobacter* in poultry-associated matrices using modified blood-free Bolton broth and selective plating, reporting good agreement with direct plating across carcasses, broiler ceca, feces, scald-tank water, and feed samples [75].

The main strength of MPN is its ability to estimate viable contamination when cell numbers are low, unevenly distributed, or difficult to recover by direct colony counting. However, the method is labor-intensive, consumes more media and incubation space than direct plating, and often requires subsequent confirmation of positive tubes by subculture or molecular testing. In addition, MPN estimates may have wide confidence intervals because they are statistical approximations rather than direct colony counts. Comparative studies suggest that when *Campylobacter* levels are high enough for reliable plate counting, direct plating is generally preferable because it is faster, simpler, and less labor-intensive [14,76]. Therefore, MPN should be considered a targeted enumeration strategy rather than a replacement for CFU-based counting. Its greatest value lies in low-concentration or matrix interference-prone samps where viable estimates are needed, but direct plating is unlikely to perform well.

### 4.3. Immunological Methods

Immunological methods detect *Campylobacter* antigens rather than relying on bacterial growth. The main formats used in this field are enzyme-linked immunosorbent assays, such as ELISA or Enzyme Immunoassay (EIA), and lateral-flow immunochromatographic assays. Their major advantage is speed, as the analytical step can often be completed within minutes to a few hours; however, they are best considered screening tools rather than definitive methods because they do not provide isolates, usually do not distinguish viable from non-viable cells, and often require enrichment when antigen concentrations in food samples are low. Enrichment improves assay sensitivity by increasing bacterial numbers and, consequently, the concentration of detectable *Campylobacter* antigens. ELISA-based methods have shown value for the rapid detection of thermotolerant *Campylobacters* in poultry products and clinical specimens. A commercial automated ELISA was reported to be repeatable and reliable for detecting thermophilic *Campylobacters* in naturally contaminated poultry products [77]. In clinical testing, EIAs have demonstrated high sensitivity and specificity in controlled validation studies, but routine use may yield false positives and lower positive predictive value, underscoring the need for culture confirmation of positive results [78,79].

Lateral-flow assays offer greater simplicity and are attractive for rapid flock or near-line screening, but their performance depends strongly on bacterial load and assay design. A direct lateral-flow test in chicken feces was mainly useful for identifying high-shedding flocks, with detection requiring high *Campylobacter* levels [80]. More advanced nanoparticle-assisted lateral-flow formats have improved sensitivity and specificity in poultry samples, but these remain controlled assay systems rather than universal replacements for reference culture [81]. The key limitations of immunological methods are matrix-dependent sensitivity, potential antibody cross-reactivity, limited quantitative precision, and limited isolate recovery. False-positive responses may arise from cross-reactivity or matrix effects, particularly in complex food samples [82]. Therefore, ELISA and lateral-flow assays are most useful as rapid front-end screening tools that can prioritize samples for confirmatory testing, rather than as standalone reference methods.

## 5. Molecular Detection and Quantification Approaches

Molecular methods have become essential complements to culture-based *Campylobacter* detection, providing faster identification, greater analytical sensitivity, and improved capacity for species differentiation and quantification. These approaches are particularly valuable when cells are present at low abundance, are physiologically injured by food-processing conditions, or occur in matrices containing high background microbiota or PCR inhibitors. However, molecular detection must be interpreted carefully, as DNA-based assays do not necessarily indicate viability, infectivity, or culture recoverability. Therefore, PCR, qPCR, digital PCR, and viability-PCR are best viewed as complementary tools that extend, rather than fully replace, culture-based surveillance.

### 5.1. Conventional PCR and Multiplex PCR

Conventional PCR remains one of the most widely used molecular approaches for detecting and identifying *Campylobacter* spp. in food and clinical samples. Established assays typically combine conserved *Campylobacter* genes (such as 16S or 23S rRNA) with targets characteristic of *C. jejuni* and *C. coli*, including the *hipO*, *mapA*, *glyA*, and *ceuE* genes [23,83]. Comparative evaluations of 12 PCR assays have shown that detection performance depends strongly on the gene chosen: *cdtC* and *hipO* provided the highest accuracy for *C. jejuni*, whereas *cdtB*, *cdtA*, and *ask* performed best for *C. coli*. [84]. In routine practice, conventional PCR is often applied alongside culture, either directly on enriched samples or on bacterial isolates, to confirm the presence of *Campylobacter* and differentiate *C. jejuni* and *C. coli*. However, because conventional PCR amplifies DNA from both viable and dead cells, it cannot distinguish viable organisms or detect VBNC cells from culturable viable cells or dead cell DNA, limiting its utility for quantitative microbial risk assessment along the food production continuum.

Multiplex PCR has been widely applied for the rapid detection and differentiation of *Campylobacter* species from feces, environmental samples, carcasses, food products, and laboratory isolates by simultaneously targeting multiple species-specific genes. In bovine fecal samples, direct multiplex PCR coupled with optimized DNA extraction effectively removed PCR inhibitors and simultaneously targeted the *mapA*, *ceuE*, 23S rRNA, and 16S rRNA genes to detect multiple *Campylobacter* species [85]. A colony multiplex PCR assay was subsequently developed for the simultaneous identification of the five major clinically important *Campylobacter* species by targeting the 23S rRNA gene and *hipO* (*C. jejuni*), *glyA* (*C. coli*, *C. lari*, and *C. upsaliensis*), and *sapB2* (*C. fetus* subsp. *fetus*), enabling accurate identification of both reference strains and clinical isolates with high specificity, including mixed cultures [86]. Species identification was further improved by a *lpxA*-based multiplex PCR, which explained species-specific sequence variation within the housekeeping gene *lpxA* to accurately differentiate *C. jejuni*, *C. coli*, *C. lari*, and *C. upsaliensis* from clinical, animal, and environmental isolates [87]. For subspecies identification, a *napA*/*napB*-based multiplex PCR identified *C. jejuni* subsp. *jejuni* and *C. jejuni* subsp. *doylei* by targeting characteristic deletions within the nitrate reductase locus [88].

Multiplex PCR combined with high-resolution melting (HRM) analysis further enhanced species differentiation by targeting the *hipO* gene of *C. jejuni* and the *asp* gene of *C. coli*. The assay distinguished the two species based on their melting profiles, detected intraspecies sequence variation, was reported achieved 100% sensitivity and 92% specificity, and produced results within approximately 8 h, demonstrating its utility for rapid speciation of clinical and poultry isolates [89]. It has also been successfully applied to environmental samples. Following selective enrichment, multiplex PCR targeting 16S rRNA, *mapA*, and *ceuE* efficiently detected *Campylobacter* from slaughterhouse surfaces, equipment, and wash-water areas, with detection rates approaching those of commercial PCR platforms after 48 h enrichment and showing strong agreement with culture-positive samples [90]. Similarly, a food-based multiplex PCR assay generated a 159 bp species-specific amplicon for *C. jejuni* and accurately detected the organism in dairy products and in raw and ready-to-eat foods. The assay showed complete agreement with conventional microbiological methods while reducing the detection time to approximately 8 h, highlighting its potential as a rapid tool for foodborne *Campylobacter* detection [91].

### 5.2. Quantitative PCR (qPCR)

qPCR enables real-time monitoring of DNA amplification, allowing quantitative assessment of contamination in diverse matrices, including poultry feces, litter, carcass rinses, meat products, milk, water, and environmental samples. In poultry fecal and cecal samples, complex sample matrices and PCR inhibitors can limit the accuracy and sensitivity of molecular detection. Therefore, effective cell-concentration and DNA-extraction procedures are important to separate target *Campylobacter* cells from inhibitory matrix components before qPCR analysis. Multiplex qPCR assays have expanded analytical capacity by simultaneously detecting and differentiating multiple *Campylobacter* species within a single reaction. For example, probe-based multiplex qPCR assays targeting species-specific genes, including *hipO* for *C. jejuni*, *glyA* for *C. coli*, and *pepT* for *C. lari*, achieve highly accurate reaction efficiencies for identifying these bacteria: 90.85% for *C. jejuni*, 96.97% for *C. coli*, and 92.89% for *C. lari*. The assay is highly sensitive, detecting as few as 10 genome copies per reaction. More recently, multiplex qPCR has been expanded beyond pathogen detection to include simultaneous identification of AMR determinants. Numerous assays have been developed targeting species-specific genes such as *mapA*, *hipO*, *ceuE*, and 16S rRNA, enabling accurate identification and quantification of major thermophilic *Campylobacter* species [92,93,94]. However, increasing the number of targets in multiplex qPCR increases assay complexity, and competitive amplification among targets can reduce amplification efficiency and analytical sensitivity, particularly when multiple targets are amplified simultaneously [95,96].

**Figure 3:**
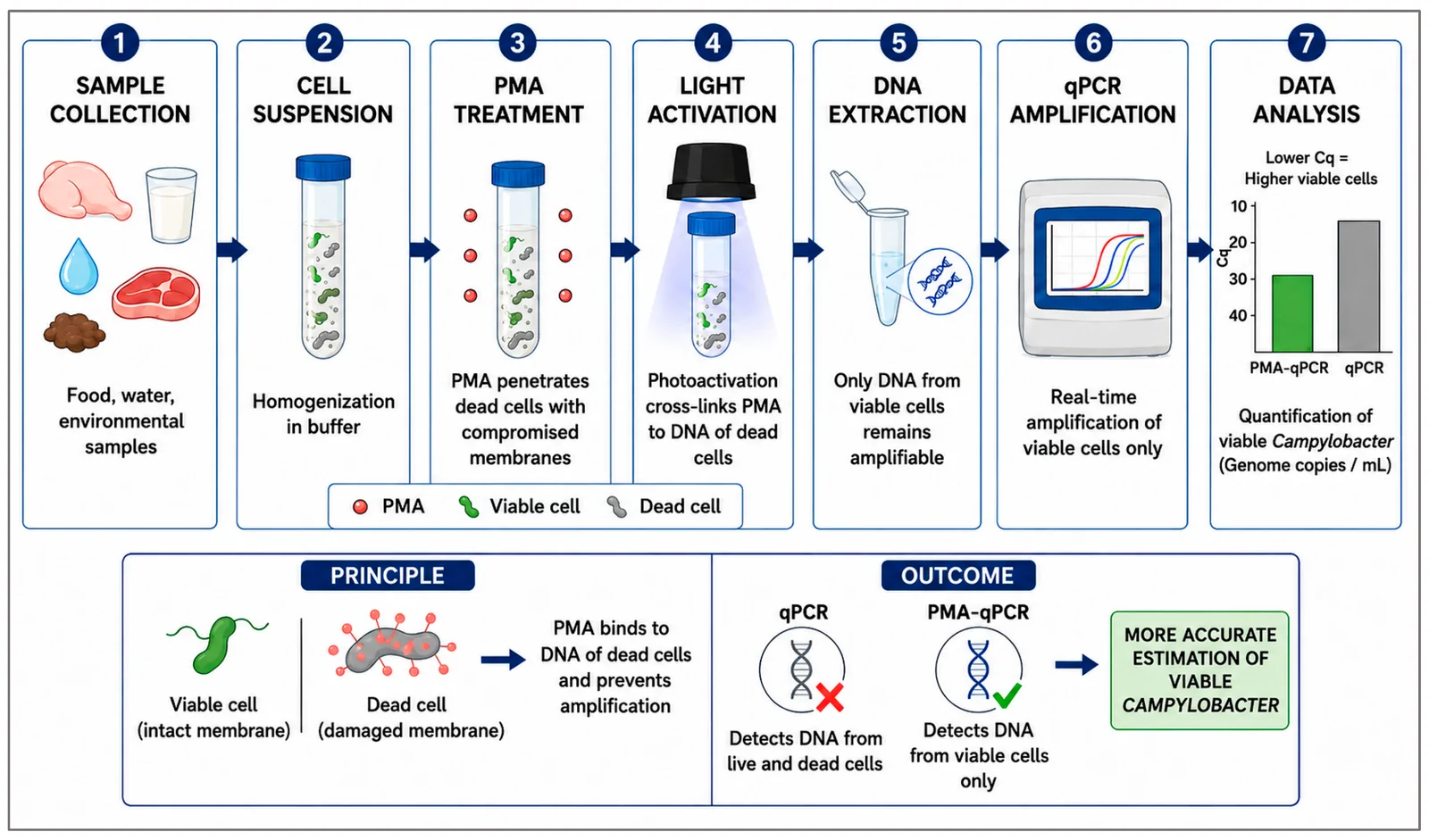
The propidium monoazide (PMA)-qPCR workflow for viability-informed detection of *Campylobacter*. The schematic illustrates the major steps of PMA-qPCR, including sample collection, cell suspension, PMA treatment, light activation, DNA extraction, qPCR amplification, and data analysis. PMA selectively penetrates membrane-compromised dead cells and, after photoactivation, binds their DNA to prevent amplification. As a result, PMA-qPCR preferentially detects viable or membrane-intact *Campylobacter* cells and provides a more accurate estimate of viable contamination than conventional qPCR (Created in BioRender. Bommineni, V. (2026) https://BioRender.com/cj2sg8s).

Among these approaches, propidium monoazide (PMA)-qPCR is the most widely used viability-informed qPCR strategy. PMA enters membrane-compromised dead cells and, following photoactivation, covalently binds DNA and prevents its amplification, thereby enriching the signal from viable or membrane-intact *Campylobacter* cells. However, PMA-qPCR performance is highly matrix-dependent and is influenced by dye concentration, light exposure, organic matter, turbidity, and sample-processing conditions. The principle and workflow of PMA-qPCR for viability-informed *Campylobacter* detection are summarized in Figure 3. Similarly, reverse transcriptase qPCR (RT-qPCR), which targets mRNA rather than DNA, provides a more reliable indicator of bacterial viability and metabolic activity, although RNA stability and extraction efficiency remain important limitations. These approaches have demonstrated the presence of VBNC *Campylobacter* populations in poultry production environments and food samples, highlighting contamination that would be overlooked by culture-based methods [38,73,97,98].

### 5.3. Digital PCR

Digital PCR (dPCR) is an advanced PCR technology that enables absolute quantification of target nucleic acids by partitioning a sample into a large number of independent reactions and applying Poisson statistics, thereby improving quantitative accuracy, including those present in complex food matrices [99]. In broiler neck-skin samples, both dPCR and qPCR showed strong correlations with ISO plate counts for *C. jejuni*, and dPCR offered greater tolerance to variation in amplification efficiency and PCR inhibitors, highlighting its potential as a complementary tool for routine monitoring and regulatory applications [100]. Droplet digital PCR (ddPCR) has also been optimized for complex tissues such as chicken liver, where PCR inhibitors frequently reduce detection accuracy. Optimized sample preparation, including improved tissue homogenization and filtration, substantially reduced matrix interference, resulting in droplet quality comparable to that of pure cultures. These findings emphasize that appropriate sample preparation is as important as the ddPCR chemistry for achieving reliable *Campylobacter* quantification in inhibitor-rich tissues [101]. dPCR has also been combined with PMA to selectively detect viable *Campylobacter*, including VBNC cells that often escape conventional culture methods. Compared with PMA-qPCR, PMA-ddPCR demonstrated superior sensitivity for detecting viable *C. jejuni* while effectively suppressing signals from dead cells. This represents an important advance because VBNC *Campylobacter* can persist in poultry-associated matrices, making PMA-ddPCR a promising tool for more accurate food safety monitoring [102].

### 5.4. Viability PCR

Viability PCR (vPCR) is an umbrella term for PCR-based approaches that use DNA-intercalating dyes, such as PMA, enhanced PMA (PMAxx), or ethidium monoazide (EMA), to reduce amplification from membrane-compromised dead cells. In contrast to conventional PCR or qPCR, which amplify DNA from both live and dead bacteria, vPCR preferentially detects viable or membrane-intact *Campylobacter* populations. PMA-qPCR is therefore a specific vPCR format that combines PMA treatment with quantitative PCR, whereas PMA-ddPCR applies the same viability principle to digital PCR for more sensitive absolute quantification.

Recent studies have optimized a PMAxx-based vPCR workflow for detecting viable *Campylobacter* in raw milk and water by systematically evaluating sample concentration and DNA extraction methods before PMAxx treatment [103]. The optimized assay demonstrated excellent linearity with detection limits ranging from 4.67 × 10^3^ to 9.84 × 10^1^ CFU/mL for *Campylobacter* [103]. In addition, PMA combined with digital PCR has further improved viability detection by enabling sensitive quantification of VBNC *C. jejuni* without a PMA enhancer, highlighting the potential of PMA-ddPCR for improving the detection of viable *Campylobacter* in food safety applications [102]. Despite these advantages, vPCR should be interpreted as a membrane-integrity-based estimate rather than a direct measure of infectivity. Dye penetration and signal suppression can vary with sample matrix, turbidity, organic load, cell density, dye concentration, and photoactivation conditions. Several approaches have been evaluated to improve PMA-based viability discrimination in complex *Campylobacter* samples. In chicken meat, two-round PMA treatment has been shown to more effectively suppress qPCR signals from dead *Campylobacter* cells than a single treatment, and PMAxx combined with PMA Enhancer has also been evaluated to improve discrimination between viable and dead cells [104]. In addition, sample pre-treatment has been used to reduce matrix interference; for example, centrifugation has been applied to remove larger debris and organic material before PMA-based analysis [38,105]. However, PMA performance remains dependent on the sample matrix and experimental conditions, and studies of broiler carcasses have reported incomplete suppression of signals from dead cells [97]. Accordingly, these approaches should be regarded as matrix-specific optimization strategies rather than as a universally standardized PMA protocol for poultry samples. vPCR is best used as a complementary approach alongside culture, qPCR, dPCR, and, conventional recovery methods when bacterial isolates are required for downstream applications.

## 6. Advanced and Emerging Technologies

Advanced and emerging technologies are increasingly being explored to overcome key limitations of conventional *Campylobacter* detection, including slow turnaround times, limited sensitivity for stressed or VBNC cells, and the need for isolate recovery prior to downstream characterization. These approaches include whole-genome sequencing, shotgun metagenomics, biosensors, microfluidic and lab-on-a-chip platforms, and artificial intelligence/machine learning-assisted analytics. Rather than replacing culture-based methods, these technologies expand the surveillance toolkit by improving strain-level resolution, source attribution, AMR profiling, rapid screening, and data integration across the food production continuum. However, their routine application in food-safety monitoring still requires further validation, standardization, cost reduction, and matrix-specific performance evaluation.

### 6.1. Whole-Genome Sequencing (WGS)

**Figure 4:**
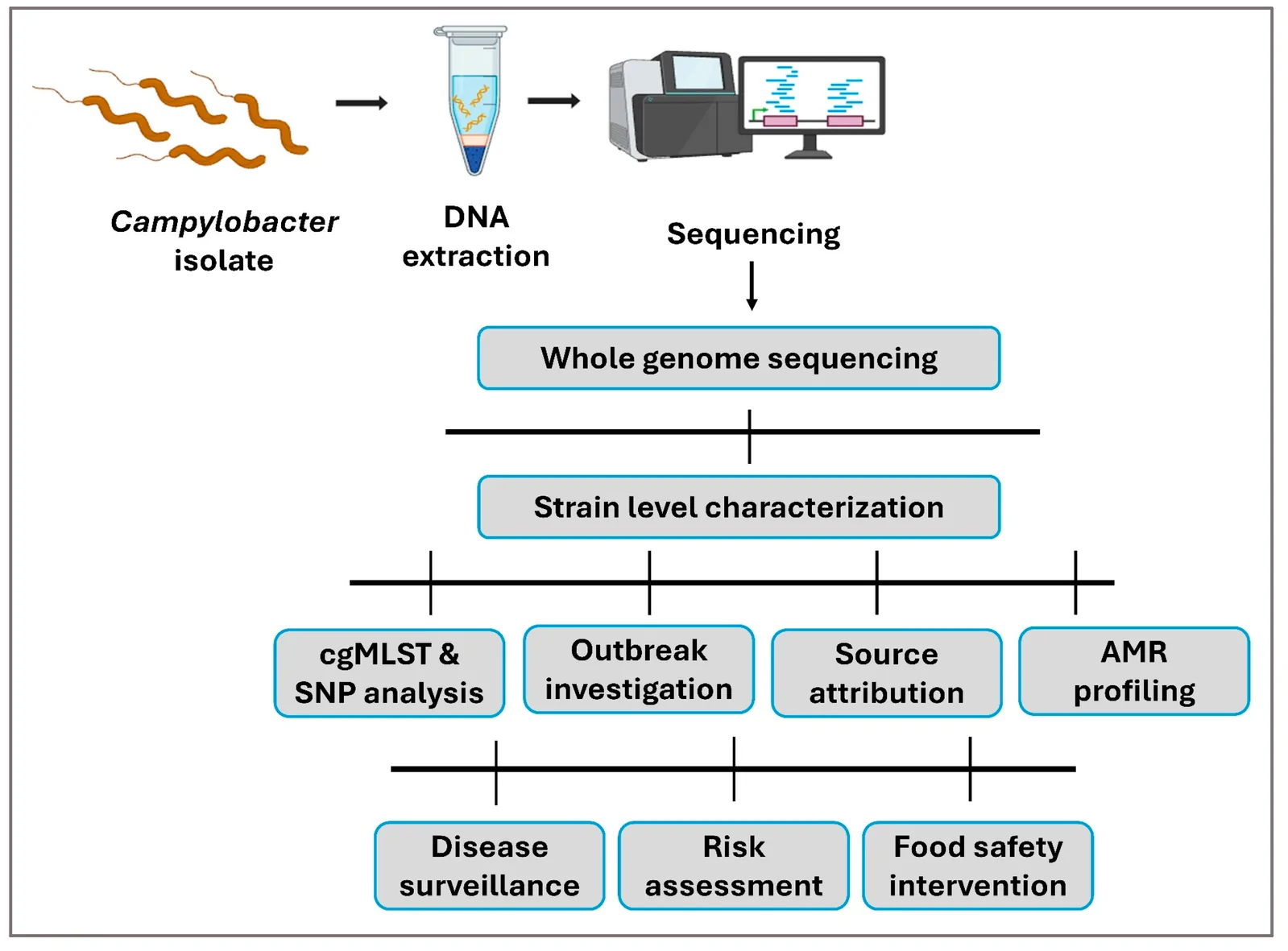
Whole-genome sequencing workflow for *Campylobacter* surveillance and source attribution. A viable *Campylobacter* isolate undergoes DNA extraction, whole-genome sequencing (WGS), and strain-level characterization. Downstream analyses, including cgMLST and SNP analyses, outbreak investigation, source attribution, and antimicrobial-resistance (AMR) profiling, support disease surveillance, risk assessment, and food-safety intervention across the food production continuum (Created in BioRender. Bommineni, V. (2026) https://BioRender.com/l0bnr5l).

Whole-genome sequencing (WGS) has become an important tool for *Campylobacter* surveillance across the food production chain, primarily for detailed characterization of isolates rather than for routine detection or quantification. As illustrated in Figure 4, WGS-based *Campylobacter* surveillance begins with recovery of a viable isolate, followed by DNA extraction, sequencing, and downstream strain-level analyses. These analyses support core genome multilocus sequence typing (cgMLST) and single-nucleotide polymorphism (SNP)-based comparisons, outbreak investigation, source attribution, AMR profiling, disease surveillance, risk assessment, and food-safety intervention. At the farm and processing stages, cgMLST and SNP phylogeny are being used to resolve flock-associated lineages, recurrent introductions, and slaughterhouse cross-contamination [106]; abattoir-based WGS studies from Europe and North America using longitudinal sampling in chicken abattoirs have revealed repeated upstream reintroduction of specific lineages and probable between-batch transmission [107]. Similarly, multi-year abattoir surveillance has detected persistent *C. jejuni* ST-21 and related clones together with recurrent AMR determinants such as *tet*(O), *blaOXA*, and *gyrA* T86I in broiler populations and carcasses [108]. Studies from Denmark demonstrated that routine WGS could identify previously unrecognized *C. jejuni* outbreaks and accurately match patient isolates with food and environmental sources [109]. WGS analysis of milk-borne outbreaks showed that epidemiologically linked isolates exhibited minimal genomic variation, allowing precise confirmation of outbreak clusters [110]. Comparative genomic studies in France further identified poultry and ruminants as the major reservoirs contributing to human campylobacteriosis [111]. In addition, WGS confirmed the epidemiological links identified by PFGE, clustering the outbreak-associated *C. jejuni* isolates into a single genomic group associated with contaminated chicken liver [112].

Beyond outbreak investigations, WGS has been widely applied to characterize *C. jejuni* and *C. coli* isolates recovered from foodborne outbreaks and poultry sources. WGS-based surveillance in Denmark has shown that routine sequencing of clinical and food isolates can uncover numerous previously unrecognized *C. jejuni* outbreaks linked to chicken meat and allow precise matching of patient isolates to retail and production sources, thereby enhancing the sensitivity and specificity of outbreak detection [109]. WGS has been applied to characterize *C. jejuni* and *C. coli* isolates from foodborne outbreaks and poultry sources [113]. Sequencing of *C. coli* YH502 from retail chicken identified a plasmid-borne type VI secretion system along with virulence-associated and AMR genes [114]. Similarly, complete genome sequencing of three chicken-derived *C. jejuni* strains revealed that they carried megaplasmids carrying the tetracycline resistance gene *tet*(O) [115].

### 6.2. Shotgun Metagenomic Sequencing

Shotgun metagenomic sequencing has been evaluated for the detection and characterization of *Campylobacter* in poultry-house air samples. In a pilot study, air sampling combined with shotgun metagenomic sequencing detected *Campylobacter* at concentrations as low as 200 CFU per sample in spiked air filters and identified *Campylobacter* in air samples collected from naturally contaminated poultry houses. The study also showed that shotgun metagenomics detected diverse microbial communities in poultry-house air, including genera that harbor opportunistic pathogenic species. However, the authors reported that the method is affected by DNA extraction bias, laboratory contamination, and challenges in taxonomic classification, indicating that careful data interpretation and further optimization are required before routine application [116].

### 6.3. Biosensors

Biosensors represent an emerging class of analytical tools for the detection and quantification of *Campylobacter* across the food production continuum [117]. By integrating selective recognition elements (such as antibodies, aptamers, or nucleic acid probes) with transducers that convert binding events into measurable electrical, optical, or piezoelectric signals [24,118], recent developments include electrochemical, optical, piezoelectric, nanoparticle-based, and CRISPR-assisted platforms. The biosensor platforms developed for *Campylobacter* detection vary considerably in their biosensor type, recognition targets, sample matrices, and analytical performance. A detailed comparison of representative biosensors, including their biosensor type, recognition target, sample matrix, limit of detection (LOD), and references, is provided in Table 1. Although few platforms have yet progressed to commercial deployment for *Campylobacter* in foods, ongoing work demonstrates their potential to support continuous or at-line surveillance, reduce dependence on culture-based methods, and enable earlier intervention points in the farm-to-fork chain.

### 6.4. Microfluidics and Lab-on-a-Chip

Microfluidic technologies have emerged as promising platforms for rapid *Campylobacter* detection by integrating multiple analytical steps into compact lab-on-a-chip devices. Ma et al. developed a polymer-based microfluidic device for the simultaneous identification and antimicrobial susceptibility testing (AST) of *Campylobacter* spp. using chromogenic media. The device achieved 100% specificity for *Campylobacter* identification and detected *C. jejuni*, *C. coli*, and *C. lari* in artificially contaminated milk and poultry meat with detection limits of 1 × 10^2^ CFU/mL and 1 × 10^4^ CFU/25 g, respectively. In addition, on-chip AST showed 91–100% agreement with the conventional agar dilution method and reduced the overall analysis time to 24 h for presumptive colonies compared to standard methods [131]. More recently, Chen et al. developed a hybrid paper/polymer-based microfluidic device integrating paper-based DNA extraction, recombinase polymerase amplification (RPA), and lateral flow detection for point-of-need detection of *C. jejuni*. The assay demonstrated 100% specificity toward *C. jejuni* and achieved a detection limit of 46 CFU/mL using cellulose paper-based DNA extraction, while the integrated microfluidic device detected 460 CFU/mL. In spiked chicken meat, the device detected *C. jejuni* at 10^1^–10^2^ CFU/g following 5–10 h enrichment, whereas samples containing more than 10^2^ CFU/g were detected without enrichment. By integrating DNA extraction, isothermal amplification, and visual detection into a single portable platform, the device provides a simplified sample-to-answer workflow suitable for on-site food testing [132].

In addition to paper-based platforms, electrochemical microfluidic systems have also been investigated for *Campylobacter* detection. Morant-Miñana et al. developed an electrochemical microfluidic biosensor based on thin-film gold microelectrodes fabricated on cyclo olefin polymer (COP) substrates for the detection of *Campylobacter* spp. The device combined PCR amplification with electrochemical detection and demonstrated a linear response between 1 and 25 nM PCR amplicons, with a limit of detection of 90 pM. The platform successfully detected *Campylobacter* in poultry meat samples. The authors proposed this biosensor as a key component of a future lab-on-a-chip platform integrating sample preparation, PCR amplification, and electrochemical detection for rapid diagnostics of foodborne pathogens [133]. For retail and field applications, cost and storage stability remain important considerations for the practical implementation of microfluidic assays. Although microfluidic platforms can substantially reduce reagent consumption, the cost of scalable manufacturing and routine replacement of disposable chips must be considered for high-throughput food testing. In addition, reagent stability may limit deployment where refrigerated storage is unavailable. In a microfluidic-based RPA device, air-dried reagents maintained the same detection sensitivity for up to 12 h at room temperature, whereas lyophilized reagents stored at −20 °C retained comparable sensitivity for 3 days, however, detection sensitivity decreased approximately 10-fold after 7–25 days of storage. These findings indicate the need for cost-effective disposable devices and improved reagent stability at room temperature before routine retail and field deployment is feasible [132]. Collectively, these studies demonstrate that microfluidic platforms are evolving from proof-of-concept devices toward integrated sample-to-answer systems that combine sample preparation, nucleic acid amplification, and detection within a single portable device. As the integration of multiple analytical functions into a single platform continues to improve, these technologies have the potential to support rapid, sensitive, and routine *Campylobacter* detection for food safety applications.

### 6.5. AI and Machine Learning Integration

Artificial intelligence (AI) and machine learning (ML) are emerging as valuable tools for enhancing *Campylobacter* surveillance, source attribution, and detection across the food production continuum. Rather than replacing conventional microbiological methods such as culture, PCR, or WGS, AI algorithms complement these techniques by extracting meaningful patterns from large and complex datasets. In large-scale national surveillance studies, machine learning models have successfully analyzed thousands of *Campylobacter* genomes to identify poultry as the predominant source of human infection and to monitor the emergence and dissemination of AMR, demonstrating the potential of AI-assisted genomic epidemiology for evidence-based food safety interventions [134]. Recent studies have demonstrated that ML integrated with WGS and cgMLST substantially improves the attribution of human *Campylobacter* infections to their animal reservoirs, enabling more accurate identification of transmission sources and supporting foodborne disease surveillance [135].

Beyond genomic source attribution, machine learning has been explored for *Campylobacter* detection. Zhang et al. [136] developed an ML-assisted spectrophotometric approach (MPN-Spectro-ML) in which absorbance spectra collected from enriched water samples were analyzed using support vector machine, logistic regression, and random forest models to predict *Campylobacter* presence. The models achieved an average prediction accuracy of approximately 76%, with relatively low false-negative rates, indicating that inexpensive spectrophotometric measurements combined with AI can enable rapid preliminary screening before confirmatory molecular testing. Although quantitative estimation of bacterial concentration remained less accurate than conventional MPN-PCR, the study demonstrated the potential of AI-assisted optical sensing to shorten detection time and reduce analytical costs, particularly in resource-limited settings [136].

ML approaches have also been applied to predict pre-harvest *Campylobacter* prevalence and associated risk factors. Using data collected from 11 pastured-poultry farms, researchers developed random forest models to predict *Campylobacter* prevalence in fecal and soil samples and identify farm-management factors associated with its occurrence. The type of farm-animal feces was the most important predictor of fecal *Campylobacter* prevalence, whereas soy-containing brood feed was associated with a higher probability of *Campylobacter* isolation from soil [137].

In a separate study, ML integration of poultry microbiome profiles with farm-management practices identified inverse associations between *Campylobacter* and potential probiotic populations. In particular, *Bacillus* and *Clostridium* were negatively correlated with *Campylobacter* at the mid-production sampling point, while potential probiotic taxa were negatively associated with *Campylobacter* at early and late production stages. These models further predicted that management factors, including frequency of pasture-house movement, age at pasture introduction, diet composition, and the presence of other animal species, could influence microbial populations associated with *Campylobacter* prevalence [138]. Together, these studies demonstrate the potential value of integrating biological and farm-management data into predictive pre-harvest surveillance. However, the reported associations should be interpreted as hypothesis-generating and validated across production systems and geographic regions before they are used as prescriptive on-farm interventions.

## 7. Quantification Challenges Across the Food Chain

Accurate quantification of *Campylobacter* along the food production continuum is constrained by the organism’s biology, the complexity of food matrices, and the limitations of both culture-based and molecular methods.

### 7.1. Viability and Methodological Limitations

The challenge is that *Campylobacter* enters VBNC states under cold storage, oxidative stress, and other environmental pressures, leading to metabolically active, potentially infectious cells that fail to grow on routine culture media. As a result, culture-based enumeration underreports true loads on carcasses and retail products, particularly late in the cold chain, while qPCR frequently yields higher results by detecting DNA from both viable and dead cells [139,140]. Viability-qPCR (PMA/EMA-qPCR) and PMA-dPCR partly bridge this gap by suppressing signal from membrane-compromised cells [73], but dye performance is strongly matrix-dependent, and single-round PMA often fails to fully remove dead-cell DNA in poultry matrices [97], making viability discrimination difficult.

### 7.2. Matrix Effects and Sampling-Matrix Differences

Matrix effects and sampling heterogeneity introduce additional uncertainty in *Campylobacter* quantification along the broiler production chain. Complex poultry matrices, such as neck skin, carcass rinses, feces, cecal contents, and meat cuts, can contain PCR inhibitors that reduce DNA extraction efficiency and amplification performance, so unoptimized workflows may underestimate *Campylobacter* levels even when using sensitive qPCR or dPCR assays [141,142]. Comparative studies have shown that different sampling schemes (e.g., neck skin, breast fillet, leg meat, and cecal contents) yield systematically different *Campylobacter* distributions and that neck-skin counts correlate with but do not identically reflect loads on other carcass parts, complicating direct comparison of results across processing stages [143]. For example, in paired post-chill broiler samples, neck-skin enumeration yielded significantly higher *Campylobacter* counts than whole-carcass rinsate (2.04 log10 CFU/g versus 1.31 log10 CFU/mL, respectively) [47]. In a longitudinal study of 55 commercial broiler flocks, boot socks, drag swabs, and fecal samples showed similar sensitivities for flock-level *Campylobacter* detection and were more strongly associated with carcass-rinse loads than litter samples; boot sock and fecal sample loads were also significant predictors of post-chill carcass rinse loads [138]. However, direct quantitative equivalence among neck skin, carcass rinsate, boot socks, and retail package exudate has not been established using a standardized same-batch experimental design, highlighting the need for controlled multi-matrix comparison studies.

### 7.3. Flock-Level and Processing-Stage Variability

At the flock and slaughterhouse levels, intermittent shedding, heterogeneous colonization, and process-driven cross-contamination during defeathering, evisceration, and chilling create substantial spatial and temporal variability in contamination patterns, which makes it challenging to design sampling plans and quantitative microbial risk assessment models that accurately capture both typical loads and high-contamination events [144,145]. To provide an overall comparison of the available approaches, Table 2 summarizes the advantages, and limitations of major *Campylobacter* detection and quantification methods, including detection limits, testing time, cost, matrix compatibility, and viability discrimination.

### 7.4. Economic Considerations Across Surveillance Settings

Economic evaluation of *Campylobacter* detection workflows should consider sampling, labor, consumables, equipment, turnaround time, confirmatory testing, and the value of results for surveillance and intervention. At slaughterhouses, qPCR and dPCR can provide more rapid quantification than conventional plate counting; however, their quantitative performance depends on bacterial concentration and assay conditions. In a comparison of qPCR, dPCR, and the ISO plate-count method in poultry samples, molecular methods showed limitations in quantification at low contamination levels (approximately 500–1000 CFU/g), indicating that culture-based enumeration remains important when standardized low-level viable counts are required [100]. At retail, culture- and PCR-based performance is matrix- and sampling-dependent, and current evidence does not establish a universally superior or cost-effective workflow. At the household level, culture-based enrichment and enumeration have been used mainly to investigate cross-contamination from raw poultry to hands, utensils, surfaces, and ready-to-eat foods, whereas evidence comparing alternative detection methods or their cost-effectiveness remains limited [146,147].

## 8. Standardization and Regulatory Frameworks

### 8.1. International Standard Methods for Campylobacter Detection and Enumeration

Standardization is essential for *Campylobacter* detection and quantification because results are used for regulatory monitoring, process hygiene assessment, outbreak investigation, AMR surveillance, and international comparisons. Since *Campylobacter* is fastidious, microaerophilic, and easily injured outside the host, differences in sampling, enrichment, selective media, incubation atmosphere, confirmation, and reporting can strongly affect measured prevalence and bacterial load. Therefore, most regulatory frameworks continue to rely on standardized culture-based methods, while molecular and genomic tools are increasingly used for confirmation, rapid screening, and source attribution [14,28,63]. The ISO 10272 series remains the principal international reference framework for food-chain testing. ISO 10272-1:2017 covers the detection of *Campylobacter* spp. by enrichment or direct plating, whereas ISO 10272-2:2017 covers enumeration by the colony count technique. The 2023 amendments are particularly important because they include molecular confirmation and identification methods for thermotolerant *Campylobacter* spp. and update culture-media performance testing, reflecting the gradual integration of molecular tools into standardized workflows [148,149].

### 8.2. Regulatory Frameworks in the European Union and United States

In the European Union, *Campylobacter* control in poultry is supported by a quantitative process hygiene criterion. Commission Regulation (EU) 2017/1495 [7] introduced a limit of 1000 CFU/g for *Campylobacter* on broiler carcasses after chilling, using the EN ISO 10272-2 method for enumeration. The sampling plan requires 50 samples, with the permitted number of samples exceeding the limit reduced over time to c = 10 from January 2025. This framework is important because it treats *Campylobacter* control as a quantitative process-hygiene issue rather than only a presence/absence hazard [7]. In the United States, *Campylobacter* monitoring is supported by FDA and USDA-FSIS methods and surveillance programs. FDA BAM Chapter 7 provides practical guidance for enrichment, isolation, and confirmation from foods, while the FSIS Microbiology Laboratory Guidebook (MLG) includes methods for isolating and identifying *Campylobacter* from poultry rinsate, sponge, and raw-product samples. FSIS also maintains a raw poultry verification program for *Salmonella* and *Campylobacter*, although current U.S. monitoring differs from the EU does not use an equivalent enforceable quantitative *Campylobacter* process-hygiene criterion for poultry products [28,55,150].

### 8.3. One Health Surveillance and Genomic Integration

Surveillance is increasingly integrated into One Health frameworks. In Europe, EFSA and ECDC publish annual One Health Zoonoses Reports, and the 2024 report identified campylobacteriosis as the most frequently reported zoonosis in humans in the EU. In the United States, NARMS tracks AMR in foodborne bacteria from humans, retail meats, and food-producing animals, while CDC PulseNet uses whole-genome sequencing for foodborne outbreak detection and has used WGS for routine *Campylobacter* surveillance since 2016 [5,151,152]. Validation of alternative methods is also critical as qPCR, dPCR, viability PCR, immunoassays, biosensors, and WGS enter surveillance workflows. ISO 16140-2 [153] provides validation principles for alternative microbiological methods against reference methods, while ISO 16140-6 addresses validation of alternative confirmation and typing methods. Laboratory competence and reproducibility are further supported by ISO/IEC 17025:2017, which defines requirements for testing-laboratory quality and technical competence [148,154,155].

Overall, current frameworks maintain culture as the reference basis for compliance, isolate recovery, and source attribution, while increasingly incorporate validated molecular and genomic tools. The major remaining gap is harmonization across biological endpoints: culture measures viable, culturable cells; qPCR measures target DNA; viability PCR estimates membrane-intact cells; dPCR improves absolute quantification; and WGS characterizes recovered isolates. Future standardization should therefore define matrix-specific sampling, analytical targets, reporting units, detection limits, viability interpretation, and metadata requirements across pre-harvest, processing, retail, and public-health surveillance.

## 9. Future Trends and Innovations

Although individual detection and typing methods each provide important information, no single approach is sufficient to capture the full epidemiological complexity of *Campylobacter* across the food production continuum.

### 9.1. Long-Term Technological Innovations

In the longer term, innovative advances in *Campylobacter* detection and surveillance are likely to arise from integrating CRISPR-based diagnostics, metagenomics and other omics approaches, and AI/ML-assisted analysis. Together, these approaches could enable increasingly culture-independent detection, characterization of complex microbial communities, and identification of contamination sources and transmission patterns from multidimensional datasets. Metagenomic surveillance is particularly promising because it can combine pathogen detection and genotyping with outbreak tracing when sequence data are interpreted alongside relevant contextual metadata. Routine adoption, however, will require extensive analytical and field validation, reduced assay costs and computational demands, harmonized data formats and metadata standards, and interoperable surveillance infrastructure [121,156]. Current public-health genomics initiatives illustrate the value of standardized sequence and contextual data for linking food, environmental, animal, and clinical isolates and for supporting rapid outbreak-source attribution.

### 9.2. Short-Term Actionable Improvements

In the short term, improvements in *Campylobacter* detection should focus on translating existing rapid and portable technologies into routine food-safety applications. Biosensors, microfluidic devices, and portable molecular diagnostic platforms could shorten detection times and enable testing closer to farms, processing facilities, and retail settings. However, their broader implementation will require standardized validation across diverse and complex food matrices, reproducible analytical performance, cost-effective disposable components, adequate reagent-storage stability, and compatibility with routine laboratory and field workflows.

Whole-genome sequencing (WGS) can also strengthen current surveillance by providing higher-resolution strain characterization, supporting AMR monitoring, and improving source attribution. For example, WGS coupled with cg MLST can identify genetic clusters; support outbreak investigation; and help distinguish likely human, food, animal, and environmental sources of *Campylobacter*. Key barriers to wider adoption include sequencing and infrastructure costs; limited bioinformatics capacity; insufficiently standardized analytical pipelines and reporting frameworks; and the need for timely, interoperable data sharing across public-health, food, animal, and environmental surveillance systems [106,107].

**Figure 5:**
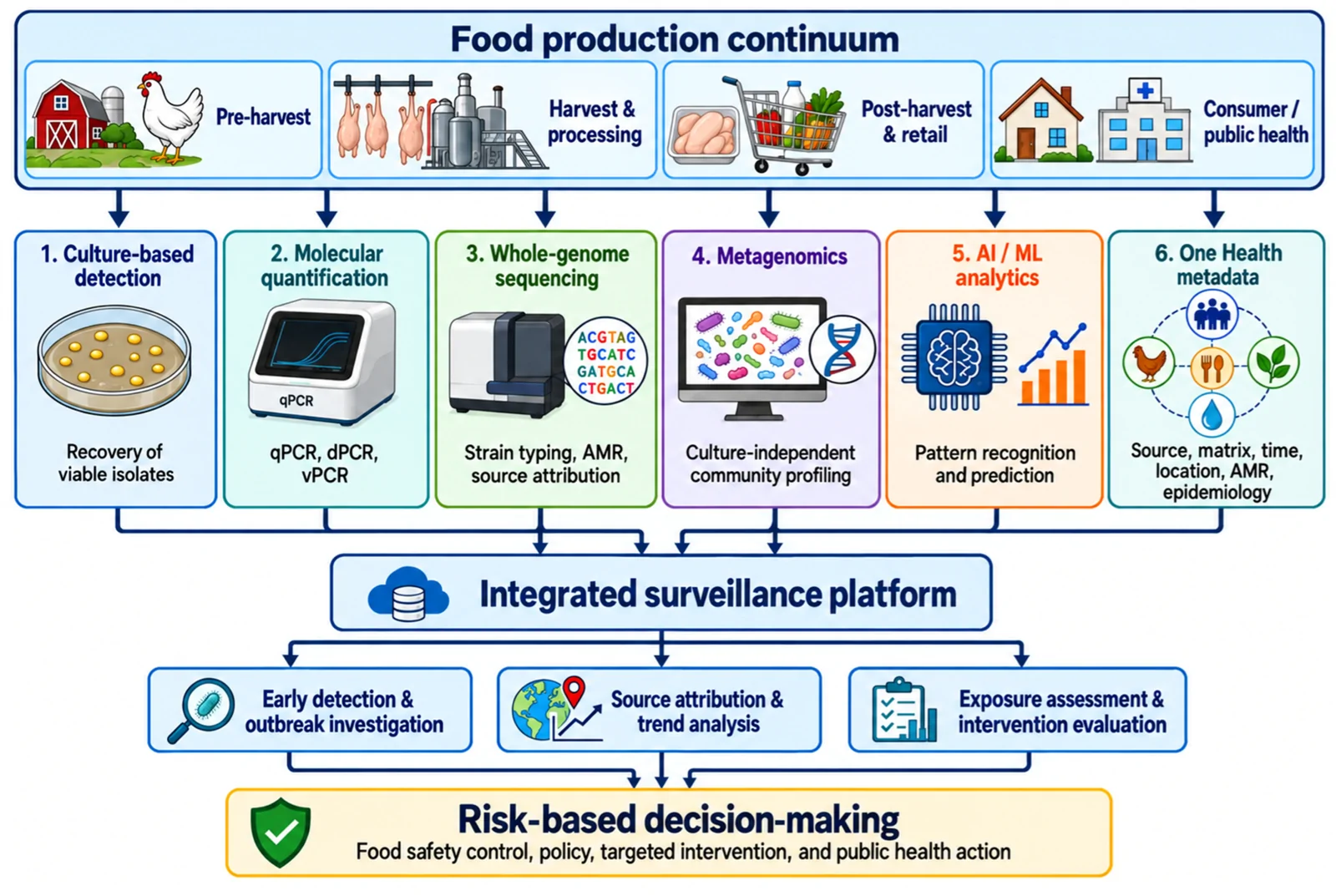
Integrated future surveillance model for *Campylobacter*. The schematic illustrates a future surveillance framework integrating culture-based detection, molecular quantification, whole-genome sequencing, metagenomics, artificial intelligence/machine learning, and One Health metadata across the food production continuum. Together, these complementary data streams support early detection and outbreak investigation, source attribution and trend analysis, exposure assessment, intervention evaluation, and ultimately risk-based decision-making for food safety and public health (Created in BioRender. Bommineni, V. (2026) https://BioRender.com/yb40840).

Metadata framework for One Health *Campylobacter* surveillance. For each sample or isolate, the framework recommends reporting (1) a unique sample/isolate ID; (2) collection date and geographic location; (3) source sector and sampling stage (farm, processing, retail, clinical, or environment); (4) sample type or matrix (e.g., feces, neck skin, carcass rinse, poultry meat, water, or human stool); (5) sampling and laboratory methods, including enrichment, detection method, and quantitative result, where available; and (6) isolate/genomic information, including species, sequencing method, strain type, and AMR results, when available [157,158]. The same core metadata fields should be reported consistently across human, animal, food, and environmental sectors to support One Health surveillance. As illustrated in Figure 5, future *Campylobacter* surveillance is likely to depend on an integrated framework that combines culture-based recovery, molecular quantification, whole-genome sequencing, metagenomics, artificial intelligence/machine learning, and One Health metadata to support risk-based decision-making. Finally, integrating human, animal, food, and environmental surveillance within a One Health framework is expected to strengthen *Campylobacter* monitoring, outbreak investigation, and evidence-based control strategies across the food production continuum.

## 10. Conclusions

This review summarizes current and emerging methods for the detection and quantification of *Campylobacter* across the food production chain and emphasizes the importance of selecting methods based on the analytical objective and sample context. Culture-based methods remain the regulatory reference for detection and enumeration and are required for the recovery, confirmation, AMR testing, and genomic characterization of isolates. However, culture-based methods can underestimate the numbers of injured, stressed, or VBNC cells. Molecular methods such as qPCR, dPCR, and viability-PCR can provide faster detection or quantification and complement culture when rapid results or viability-informed estimates are required. Their performance and interpretation, however, are still dependent on sample preparation, assay conditions, inhibition control, and the food matrix. WGS improves outbreak investigation, strain characterization, AMR monitoring, and source attribution, however conventional isolate-based workflows require successful isolate recovery. Biosensors, microfluidics, and AI-assisted approaches are also promising; however, they require further validation in naturally contaminated food matrices before routine implementation. Future work should focus on matrix-specific validation against standardized reference methods, as well as integrated workflows that combine bacterial culture, molecular quantification, and genomic data. Together, these approaches can be strengthened to support risk-based monitoring, surveillance, and control of *Campylobacter* across the food-production continuum.

## Figures and Tables

**Table 1:** Representative biosensor-based platforms for *Campylobacter* detection in food, environmental, and clinical matrices.

Biosensor Type	Recognition Element/Target	Sample Matrix	Detection Limit (LOD)	Reference
Colorimetric/SERS aptasensor	Aptamers against *C. jejuni* surface proteins	Spiked water	6 CFU/mL	[119]
Electrochemical genosensor	Thiolated DNA probe (*C. jejuni*, *C. coli*, *C. lari*, *C. upsaliensis*)	Chicken meat	1.06 pg/µL	[120]
Dual-mode RPA/CRISPR-Cas12a biosensor	*dnaA* gene	Chicken, egg, milk	0.98 pg/µL (fluorescence)	[121]
Gold nanoparticle (GNP)-based DNA biosensor	*cadF* gene	Drainage water; poultry rinse	10^3^ CFU/mL (drainage water); 10^6^ CFU/mL (poultry rinse)	[122]
Quartz crystal microbalance (QCM) immunosensor	Rabbit polyclonal antibodies; *C. jejuni*	Cell suspension	150 CFU/mL	[123]
Colorimetric aptasensor	Aptamers for *C. jejuni* and *C. coli*	Chicken carcass	7.2 × 10^5^ CFU/mL (*C. jejuni*), 5.6 × 10^5^ CFU/mL (*C. coli*)	[124]
QCM immunosensor with magnetic nanobeads	Monoclonal and polyclonal antibodies	Poultry	20–30 CFU/mL	[125]
Surface plasmon resonance (SPR) based immunosensor	Rabbit polyclonal antibody; *C. jejuni*	Chicken	131 ± 4 CFU/mL	[126]
Gold nanorod (GNR)-based DNA nanobiosensor	*cadF* gene probes	Human stool	10^2^ copy number/mL	[127]
Fluorescence immunosensor	Polyclonal antibodies	Poultry liver	10 CFU/mL	[128]
Metal oxide semiconductor (MOS) biosensor	Whole bacterial cells	Milk	10^5^ CFU/mL	[129]
DNA dot blot biosensor	CampyP3 DNA probe	Chicken meat	3 pg/mL	[130]

**Table 2:** Comparison of current and emerging methods for *Campylobacter* detection and quantification across the food production continuum.

Method	Representative Reported LOD/LOQ *	Testing Time	Relative Cost ^	Matrix Compatibility	Viability Discrimination Capacity	Advantages	Limitations
Culture-based	Method- and matrix-dependent	24–48 h enrichment + plating/confirmation	Low–moderate	Chicken meat, carcass rinses, food/environmental samples	Yes (culturable cells)	Regulatory reference method; recovers viable-culturable isolates for confirmation, antimicrobial-susceptibility testing, and WGS	Slow (typically 48–72 h or longer) and may miss injured or VBNC cells that do not grow on selective media
Immunological methods	5 × 10^4^–3.2 × 10^6^ CFU/mL (ELISA); ≥6.7–7.3 log CFU/g (direct LFA)	<15 min–3 h; up to ~2 h for direct fecal LFA	Low–moderate	Poultry products, chicken feces/cecal contents, poultry samples, food and clinical specimens	No	Simple, rapid, and potentially useful for high-throughput or on-site screening	Often require enrichment; may show matrix-dependent sensitivity or cross-reactivity and do not provide isolates or reliable viability information
Conventional PCR/Multiplex PCR	10 CFU/2 g after 16 h enrichment; 0.017 ng/PCR analytical LOD	~8 h	Moderate	Bovine feces; chicken swabs; slaughterhouse environmental samples; dairy products; raw and ready-to-eat foods; clinical, animal and environmental isolates	No	Rapid confirmation and species differentiation; multiplex formats detect several *Campylobacter* targets in one reaction	Does not distinguish viable from dead cells; sensitivity can be affected by DNA extraction efficiency and matrix-associated PCR inhibitors
Quantitative PCR/multiplex Quantitative PCR	4.6 × 10^2^ cells/mL; 4.87–16.10 genome copies/reaction; 100 fg/µL DNA	~2–4 h	Moderate	Chicken meat, chicken wing rinse, chicken juice, fried chicken strips, chicken and sheep meat	No	Rapid, sensitive detection and relative quantification; can simultaneously detect and differentiate species	Detects DNA from both viable and dead cells; results depend on standard curves, extraction efficiency, and matrix-associated PCR inhibition
Digital PCR	~1 genome copy equivalent/reaction	~2–4 h	High	Broiler neck skin; chicken liver	No	Absolute target quantification without a standard curve and greater tolerance of PCR inhibitors than qPCR	Expensive instrumentation and lower routine throughput; still detects DNA from dead cells unless combined with a viability dye
Viability PCR	9.84 × 10^1^–4.67 × 10^3^ CFU/mL (milk/water; study-dependent)	~3–6 h	Moderate–high	Raw milk, water	Yes	Reduces signals from membrane-compromised dead cells and can detect viable or VBNC *Campylobacter* missed by culture	Measures membrane integrity rather than infectivity; results are strongly affected by dye treatment, photoactivation, and matrix effects
Whole-genome sequencing	N/A	Few days	High	*Campylobacter* isolates from poultry, broiler carcasses, food, clinical and outbreak-associated samples	No	Provides high-resolution strain typing, source attribution, outbreak investigation, and AMR/virulence profiling	Usually requires a recovered isolate; not intended for primary detection or routine quantification
Shotgun metagenomic sequencing	200 CFU/sample (spiked poultry-house air filter; pilot study)	Few days	High	Spiked air filters; poultry-house air	No	Enables culture-independent detection of multiple organisms and characterization of genes in a sample	Limited sensitivity at low target abundance; host/background DNA, cost, and bioinformatic complexity restrict routine use
Microfluidics/Lab-on-a-Chip	46–460 CFU/mL (paper-based microfluidic device); 10^2^ CFU/mL (milk); 10^4^ CFU/25 g (poultry meat)	5–24 h	Moderate; platform-dependent	Milk, poultry meat	Platform-dependent	Integrates sample preparation, amplification, and detection in a compact, potentially portable format with shorter turnaround time	Most platforms remain at proof-of-concept stage and require validation in naturally contaminated, complex matrices
Biosensors	6 CFU/mL (SERS aptasensor); 100–150 CFU/mL (immunosensors); 0.96–1.06 pg/µL (DNA-based CRISPR/genosensor)	Minutes to a few hours	Low–moderate; platform-dependent	Water, poultry rinse, chicken meat/carcass, poultry liver, milk, egg, cell suspension, human stool	No	Potentially rapid, portable, and suitable for at-line or on-site screening	Performance may be affected by food-matrix interference; limited standardization and few systems are routinely used for food testing
Artificial Intelligence and Machine Learning Integration	N/A	NR	Moderate–high	Genomic surveillance/WGS data; human and animal *Campylobacter* isolates; enriched water samples	N/A	Identifies patterns in large surveillance, genomic, or sensor datasets and can support source attribution and rapid preliminary screening	Depends on high-quality, representative training data; not a standalone detection method and requires independent validation

* LOD/LOQ values represent examples reported in individual studies and should not be interpreted as directly comparable across methods because analytical performance varies with assay design, target, sample matrix, sample volume, enrichment, DNA extraction, and reporting units. Values are presented in the units reported by the original studies. ^ Relative cost categories provide a general comparison based on typical instrumentation, reagent, labor, and data-analysis requirements; actual costs may vary among laboratories. NR, not reported; N/A, not applicable; AMR, antimicrobial resistance; CFU, colony-forming units; ELISA, enzyme-linked immunosorbent assay; LFA, lateral flow assay; LOD, limit of detection; LOQ, limit of quantification; VBNC, viable but non-culturable; WGS, whole-genome sequencing.

## Data Availability

No new data were created or analyzed in this study. Data sharing is not applicable to this article.

## Supplementary Materials

The following supporting information can be downloaded at https://www.mdpi.com/article/10.3390/pathogens15101024/s1, File S1: Summary and Scope of Previous Reviews on *Campylobacter* Detection and Methodologies [17,18,19,20,21,22,23,24].

## Abbreviations

The following abbreviations are used in this manuscript:

AI	Artificial intelligence
AMR	Antimicrobial resistance
AST	Antimicrobial susceptibility testing
CDC	Centers for Disease Control and Prevention
CFU	Colony-forming units
cgMLST	Core genome multilocus sequence typing
COP	Cyclo olefin polymer
CRISPR	Clustered regularly interspaced short palindromic repeats
dPCR	Digital PCR
ddPCR	Droplet digital PCR
ECDC	European Centre for Disease Prevention and Control
EFSA	European Food Safety Authority
EIA	Enzyme immunoassay
ELISA	Enzyme-linked immunosorbent assay
EMA	Ethidium monoazide
ESBL	Extended-spectrum β-lactamase
EU	European Union
FDA	Food and Drug Administration
FDA BAM	Food and Drug Administration Bacteriological Analytical Manual
FSIS	Food Safety and Inspection Service
HRM	High-resolution melting
IEC	International Electrotechnical Commission
ISO	International Organization for Standardization
LOD	Limit of detection
LOQ	Limit of quantification
MALDI-TOF	Matrix-assisted laser desorption/ionization time-of-flight
ML	Machine learning
MLG	Microbiology Laboratory Guidebook
MPN	Most probable number
mCCDA	Modified charcoal cefoperazone deoxycholate agar
mRNA	Messenger RNA
NARMS	National Antimicrobial Resistance Monitoring System
PCR	Polymerase chain reaction
PFGE	Pulsed-field gel electrophoresis
PMA	Propidium monoazide
PMA-qPCR	Propidium monoazide quantitative PCR
PMA-ddPCR	Propidium monoazide droplet digital PCR
qPCR	Quantitative PCR
RPA	Recombinase polymerase amplification
rRNA	Ribosomal RNA
RT-qPCR	Reverse transcription quantitative PCR
SNP	Single nucleotide polymorphism
USDA-FSIS	United States Department of Agriculture—Food Safety and Inspection Service
VBNC	Viable but non-culturable
vPCR	Viability PCR
WGS	Whole-genome sequencing
WHO	World Health Organization
