# Supplementary material for: Current Status and Future Trends in Campylobacter Detection and Quantification Across the Food Production Continuum
Source: Pathogens. 2026 Sep 30;15(10):1024. doi: 10.3390/pathogens15101024 (PMC13708052; doi:10.3390/pathogens15101024)
Supplement: Supplementary file 1 [file pathogens-15-01024-s001.zip › pathogens-4500487-supplementary.pdf]

Supplementary File S1: Summary and Scope of Previous Reviews on *Campylobacter* Detection and Methodologies

Objective of review	Key sections	References
This article has reviewed the transmission and attribution sources as well as methodologies for the detection and virulence characterization of *campylobacter*ia	Epidemiology and disease manifestations; routes and sources of transmission; isolation and culture methods; species identification and typing; colonization and virulence factors; antimicrobial resistance.	Soto-Beltrá N, M., Lee, B. G., Amézquita-López, B. A., & Quiñones, B. (2023). Overview of methodologies for the culturing, recovery and detection of *Campylobacter*. International journal of environmental health research, 33(3), 307–323. https://doi.org/10.1080/09603123.2022.2029366
This review focuses on the existing variability in *Campylobacter* enrichment and isolation procedures used by researchers and regulatory agencies worldwide, for various matrices.	Direct plating; pre-enrichment; primary and secondary enrichment; enrichment conditions; poultry, dairy, produce, and farm-environment matrices; morphological, biochemical, and molecular identification	Harrison, L. M., Balan, K. V., Hiett, K. L., & Babu, U. S. (2022). Current methodologies and future direction of *Campylobacter* isolation and detection from food matrices, clinical samples, and the agricultural environment. Journal of microbiological methods, 201, 106562. https://doi.org/10.1016/j.mimet.2022.106562
This review highlights current advances in the	Detection of *Campylobacter* spp. by Electrochemical	Vizzini, P., Braidot, M., Vidic, J., & Manzano, M. (2019). Electrochemical and Optical Biosensors for the Detection of *Campylobacter* and Listeria:

development of biosensors for detection of *Campylobacter* spp. and Listeria monocytogenes that are the most common causes of zoonosis.	Biosensors, Detection of *Campylobacter* spp. by Optical Biosensors	An Update Look. Micromachines, 10(8), 500. https://doi.org/10.3390/mi10080500
This review discusses the standard microbiological protocols for *Campylobacter* spp. isolation and enumeration in food and water and evaluates detection media (pre-enrichment, selective enrichment, and selective plating) for their rational applications.	Standard procedures of detection and enumeration FDA protocols	Neyaz, L. A., Arafa, S. H., Alsulami, F. S., Ashi, H., Elbanna, K., & Abulreesh, H. H. (2024). Culture-Based Standard Methods for the Isolation of *Campylobacter* spp. in Food and Water. Polish journal of microbiology, 73(4), 433–454. https://doi.org/10.33073/pjm-2024-046
This review will focus on the development and application of rapid molecular methods for identifying and	Challenges to the Identification and Detection of *Campylobacter* Immune-Based Assays for *Campylobacter*, Conventional	Ricke SC, Feye KM, Chaney WE, Shi Z, Pavlidis H, Yang Y. Developments in Rapid Detection Methods for the Detection of Foodborne *Campylobacter* in the United States. Front Microbiol. 2019 Jan 23;9:3280. doi: 10.3389/fmicb.2018.03280. PMID: 30728816; PMCID: PMC6351486

quantifying *Campylobacter* in U.S. poultry	PCR, Multiplex PCR Detection, Real-Time Quantitative PCR (qPCR), Digital PCR (dPCR), Genotyping of *Campylobacter*, Next-Generation Sequencing of *Campylobacter*	
To review available approaches for monitoring *Campylobacter* throughout the poultry production chain and discuss the transition from conventional culture toward rapid molecular and genomic methods.	Culture-based monitoring; PCR and real-time PCR; molecular detection; strain characterization; monitoring along the poultry chain; future needs for rapid detection	Josefsen, M. H., Bhunia, A. K., Engvall, E. O., Fachmann, M. S., & Hoorfar, J. (2015). Monitoring *Campylobacter* in the poultry production chain—from culture to genes and beyond. Journal of microbiological methods, 112, 118–125. https://doi.org/10.1016/j.mimet.2015.03.007
To systematically evaluate the diagnostic performance and species-specific biases of culture-based methods used for detection of	Diagnostic sensitivity and specificity; direct culture; selective enrichment; Bolton broth; ISO 10272 approaches; food matrices; C. *jejuni* versus C. *coli* recovery	Meethai, C., Phothawon, P., Nale, J. Y., & Wisessombat, S. (2026). Comparative Performance and Species-Specific Recovery Biases of Culture-Based Methods for *Campylobacter* Detection in Food Products: A Systematic Review and Meta-Analysis. Veterinary Sciences, 13(5), 415. https://doi.org/10.3390/vetsci13050415

Campylobacter in food products.	bias; turnaround time and validation requirements.	
To trace the historical development of *Campylobacter* culture media and assess how selective and differential formulations influence detection and recovery.	Early blood-based media; charcoal-based media; selective agents; chromogenic media; species-recovery bias; matrix applicability; advantages and limitations of culture media; future data-guided culture formulations.	Barata, A. R., Saavedra, M. J., & Almeida, G. (2026). From Basics to Breakthroughs: A Review on the Evolution of *Campylobacter* spp. Culture Media. Microorganisms, 14(2), 498. https://doi.org/10.3390/microorganisms14020498
